# ZIP Genes Are Involved in the Retransfer of Zinc Ions during the Senescence of Zinc-Deficient Rice Leaves

**DOI:** 10.3390/ijms241813989

**Published:** 2023-09-12

**Authors:** Yangming Ma, Yanfang Wen, Cheng Wang, Ziniu Wu, Xiaojuan Yuan, Ying Xiong, Kairui Chen, Limei He, Yue Zhang, Zhonglin Wang, Leilei Li, Zhiyuan Yang, Yongjian Sun, Zhongkui Chen, Jun Ma

**Affiliations:** 1 Rice Cultivation Laboratory, Rice Research Institute, Sichuan Agricultural University, Chengdu 611130, China; 2 Crop Ecophysiology and Cultivation Key Laboratory of Sichuan Province, Chengdu 611130, China

**Keywords:** zinc deficiency, rice, ZIP, zinc ion retransfer, leaf senescence

## Abstract

Rice lacks sufficient amounts of zinc despite its vitality for human health. Leaf senescence enables redistribution of nutrients to other organs, yet Zn retransfer during deficiency is often overlooked. In this hydroponic experiment, we studied the effect of Zn deficiency on rice seedlings, focusing on the fourth leaf under control and deficient conditions. Growth phenotype analysis showed that the growth of rice nodal roots was inhibited in Zn deficiency, and the fourth leaf exhibited accelerated senescence and increased Zn ion transfer. Analyzing differentially expressed genes showed that Zn deficiency regulates more ZIP family genes involved in Zn ion retransfer. OsZIP3 upregulation under Zn-deficient conditions may not be induced by Zn deficiency, whereas OsZIP4 is only induced during Zn deficiency. Gene ontology enrichment analysis showed that Zn-deficient leaves mobilized more biological pathways (BPs) during aging, and the enrichment function differed from that of normal aging leaves. The most apparent “zinc ion transport” BP was stronger than that of normal senescence, possibly due to Zn-deficient leaves mobilizing large amounts of BP related to lipid metabolism during senescence. These results provide a basis for further functional analyses of genes and the study of trace element transfer during rice leaf senescence.

## 1. Introduction

Zinc (Zn) is an essential trace element for plant growth and development. However, Zn deficiency is widespread in crops globally, rendering it the most common micronutrient deficiency [1,2]. In addition, because Zn from the edible parts of crops is the primary source of Zn for humans, Zn deficiency in crops can lead to Zn deficiency in humans [3,4]. Therefore, understanding the absorption and distribution of Zn in rice is essential.

Zn plays a vital role in promoting the growth and development of rice and ensuring high yield and quality. However, due to the rich and complex soil resources available for rice cultivation worldwide, the distribution of the content of Zn ions in different soils varies greatly, and the ability of Zn uptake and accumulation in rice with different genotypes also varies [5,6]. This variability can potentially result in poor uptake and enrichment of Zn in rice. Moreover, rice primarily relies on its roots to absorb Zn from the soil and Zn fertilizer [7,8]. The effectiveness of metal extraction from the soil is controlled by three conditions [1,2,9]: (1) soil conditions (upland or flooded soil, pH value of soil solution); (2) soil zinc forms, mainly including (i) water-soluble Zn, including Zn^2+^ and soluble organic fractions; (ii) adsorbed and exchangeable Zn in the colloidal fraction (associated with clay particles, humic compounds, and Al and Fe hydroxides); and (iii) insoluble Zn complexes and minerals); and (3) transporter uptake. Root absorption can also be divided into three steps [10]: (1) rapid Zn absorption on the surface of root cells, which does not require energy consumption; (2) entry inside the root through the apoplast or symplast pathways; and (3) entry of the duct or tracheid through passive and active transport from the cell membrane into the root cell. In the root, Zn^2+^ is loaded from the xylem parenchyma cells into the xylem [11]. After the roots absorb Zn, it is transferred with high Zn demand [12]. Given the high demand for Zn to build and operate the photosynthetic apparatus of chloroplasts in photosynthetic plant tissues [13], apart from the content of Zn ions required for the development of the root system itself, zinc ions are mostly distributed to the leaves.

However, leaves will eventually age, which is an active process of death. Apart from enhancing plant adaptation to biotic and abiotic stresses, another important role of leaf senescence is to serve as a nutrient reservoir, transferring nutrients to developing organs or grains [14,15,16,17,18]. This process involves the regulation of multiple genes [14,19,20,21,22]. Many molecular factors involved in zinc uptake and transport to different plant parts have been identified [23,24]. Among them, the ZIP family genes play a major role. In rice (*Oryza sativa*, Os) and *Arabidopsis thaliana* (At), the zinc-regulated transporters ZRT and IRT gene families consist of 15 and 12 members, respectively, with 10 and 7 members upregulated under zinc deficiency [25,26,27,28]. Although ZIP transporters have been extensively studied, their specific physiological roles in senescent leaves are not fully understood. Additionally, zinc deficiency in senescent leaves can lead to low content of zinc ions, and it can also affect the expression of certain zinc transporter genes, potentially influencing zinc redistribution in leaves. The transfer of zinc in aging leaves mainly occurs through the phloem to new tissues [29,30], making a significant contribution to the mineral accumulation in rice grains [31]. The accumulation of zinc in grains depends on the zinc absorbed by the roots from the soil and the retransfer of zinc from aging leaves. Therefore, improving the transfer of zinc in aging leaves is crucial.

In this study, we used the fourth leaf of rice seedlings to determine the time point of Zn retransfer during senescence through ion analysis and explored critical factors of Zn retransfer during leaf senescence. In addition, we compared the growth phenotypes of Zn-deficient and control plants.

## 2. Results

### 2.1. Zn Deficiency Inhibits Rice Seedling Shoot Growth and Development

**Figure 1:**
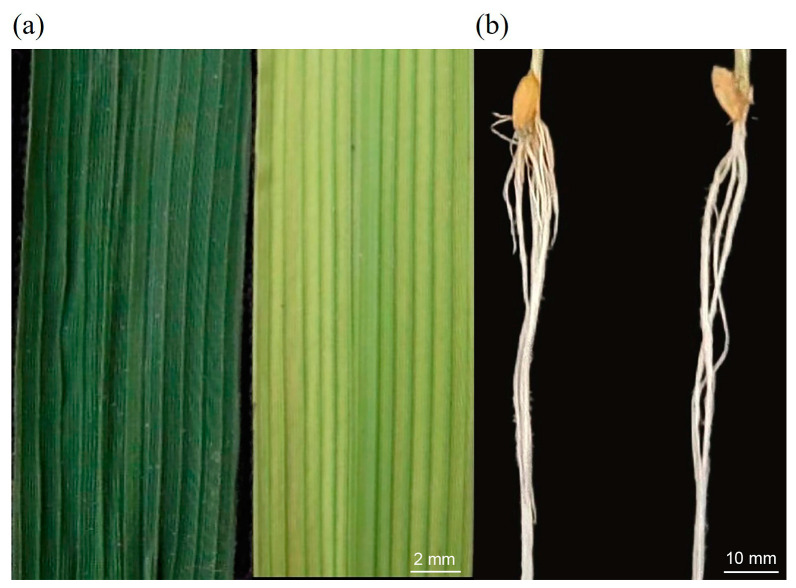
Hydroponics of rice under different zinc (Zn) concentrations. (**a**) The middle of the fourth leaf at the fourth time point under control (**left**) and Zn deficiency (**right**) conditions; (**b**) Root system under control (**left**) and Zn deficiency (**right**) conditions. The images are representative of each treated plant.

**Figure 2:**
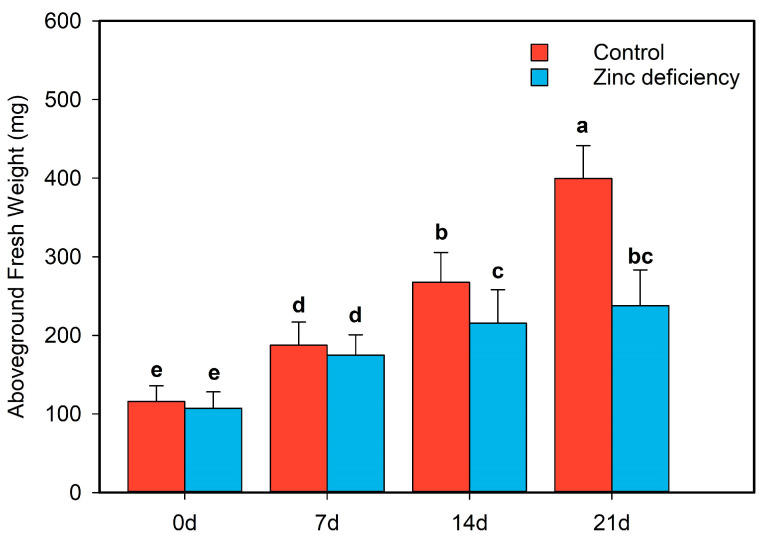
Changes of fresh weight in rice seedlings under control and zinc (Zn) deficiency treatments. The hydroponic plants were cultured under the condition of zinc deficiency (0 μM Zn) and control (0.75 μM Zn) when rice was in the two-leaf phase. The first sampling point was denoted as “0 d” when the fourth leaf was fully developed, and sampling was conducted every 7 days after that. The bar chart shows the mean values of 15 plants per treatment (+/− standard error). Statistical differences between letters according to the Student’s *t*-test (*p* < 0.05).

Zn deficiency negatively affected rice seedling shoots. The plants were slightly yellowish (Figure 1), and the shoot height was shorter than that of the control. With increased treatment time, shoot inhibition in the Zn-deficient treatment group increased, and the difference in shoot fresh weight between the Zn-deficient treatment and the control groups gradually increased. At the fourth sampling time (21 d), the shoot fresh weight of the Zn-deficient treatment group was reduced by 38.15% (Figure 2). Although the control was slightly higher than the Zn deficiency treatment at the first two time points, no notable difference in the aboveground fresh weight between the two treatments was observed. In addition, the difference in the fresh weight between the two treatment groups at the second time point was similar to that of the first time point (Figure 2).

### 2.2. Zn Deficiency Affects the Growth of the Fourth Rice Leaf

**Figure 3:**
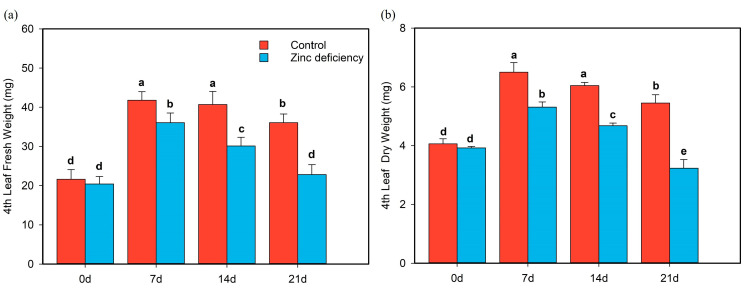
Changes in fresh and dry weight in the fourth leaf of rice seedlings for the zinc (Zn) deficiency and control conditions. The hydroponic plants were cultured under the condition of zinc deficiency (0 μM Zn) and control (0.75 μM Zn) when rice was in the two-leaf phase. The first sampling point was denoted as “0 d” when the fourth leaf was fully developed, and sampling was conducted every 7 days after that. (**a**) Fresh weight of fourth leaf and (**b**) dry weight of fourth leaf. Red all represents control, and blue all represents zinc deficiency. The bar chart shows the mean values of 15 plants per treatment (+/− standard error). Statistical difference of letters according to the Student’s *t*-test (*p* < 0.05).

Zn deficiency reduced fresh weight and dry weight of the fourth leaf, accelerated leaf senescence, and shortened the leaf growth cycle (Figure 3). At the second time point, the fresh and dry weights of the control group were considerably higher than those of the Zn-deficient treatment group, by 19.68% and 23.35%, respectively (Figure 3). On day 21, the fresh and dry weight of the fourth leaf of the control group was reduced by 16.42% and 17.98%, respectively, compared with that on day 7. Furthermore, the reduction in the Zn deficiency treatment group was greater on the 21st day, and its fresh and dry weight decreased by 36.29% and 39.24%, respectively, compared with the 7th day of the zinc deficiency group (Figure 3).

From 0 to 7 days, the biomass of the fourth leaf in both treatments increased, indicating that although the fourth leaf was fully expanded at the first sampling (0 d), it did not reach full maturity until seven days later. At the first time point (0 d), the fresh weight and dry weight of the fourth leaf in the control treatment were slightly higher than those in the zinc deficiency treatment group, but there was no significant difference between the two groups. The difference gradually increased from the second time point to the fourth time point.

### 2.3. Zn Deficiency Alters the Root Architecture of Rice Seedlings

**Figure 4:**
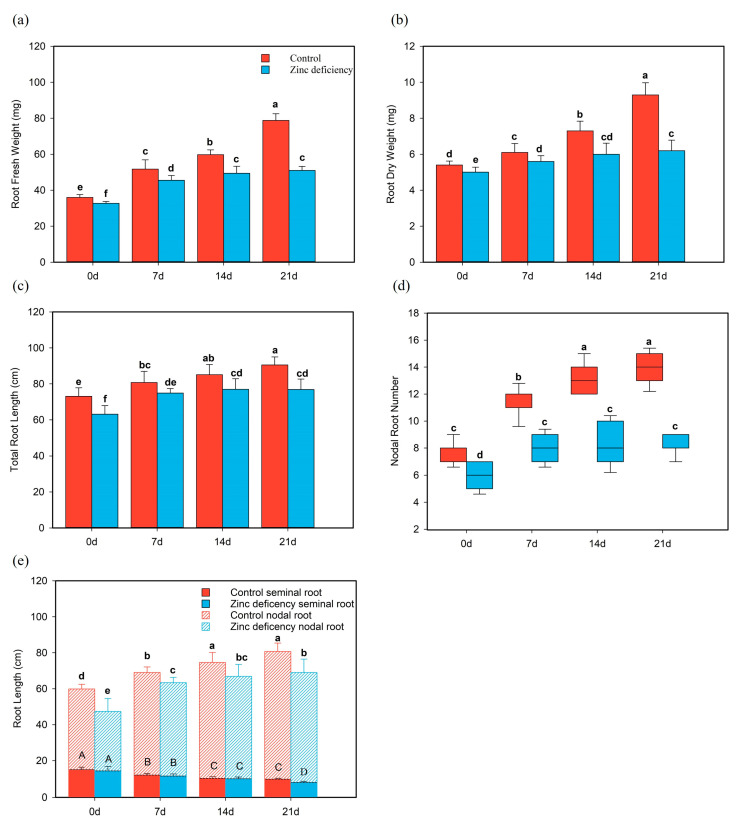
Phenotypic growth of rice seedling roots under zinc deficiency and control. The hydroponic plants were cultured under the condition of zinc deficiency (0 μM Zn) and control (0.75 μM Zn) when rice was in the two-leaf phase. The first sampling point was denoted as “0 d” when the fourth leaf was fully developed and then sampled every 7 days four times. (**a**) Fresh root weight, (**b**) dry root weight, (**c**) total root length, (**d**) number of node roots in rice seedlings, and (**e**) seminal root and node root lengths. In the figures (**a**–**d**), red all represents control, and blue all represents zinc deficiency. The bar chart shows the mean (+/− standard deviation) of the 15 plants in each treatment. Letters indicate statistical differences according to Student’s *t*-test (*p* < 0.05).

The effect of Zn deficiency on the root growth of rice seedlings differed from that on the shoot responses. Zn deficiency inhibited root elongation and development (Figure 1). The inhibition of fresh and dry root weights of Zn-deficient seedlings increased gradually. With the extension of treatment time, the inhibition of root biomass of Zn-deficient seedlings gradually increased, and the root fresh weight and dry root weight were 37.4% and 41.22% lower than that of the control at 21 days (Figure 4a,b). Similar changes were observed in the total root length of Zn-deficient rice, which was gradually inhibited by the extension of zinc deficiency time and was reduced by 13.56% compared with the control at 21 days (Figure 4c).

The difference in total root length primarily came from the node roots. At 21 days, the node root length of zinc-deficient plants was reduced by 14.53% compared with the control (Figure 4e). With the extension of treatment time, the growth of new node roots of zinc-deficient seedlings stopped. At 21 d, the number of node roots of zinc-deficient seedlings was five fewer than that of control (Figure 4d). The length of seminal roots decreased with increasing treatment time in both zinc-deficient and control plants (Figure 4e).

### 2.4. Zn Deficiency Affects Zn Ion Transfer during Senescence in the Fourth Rice Seedling Leaf

**Figure 5:**
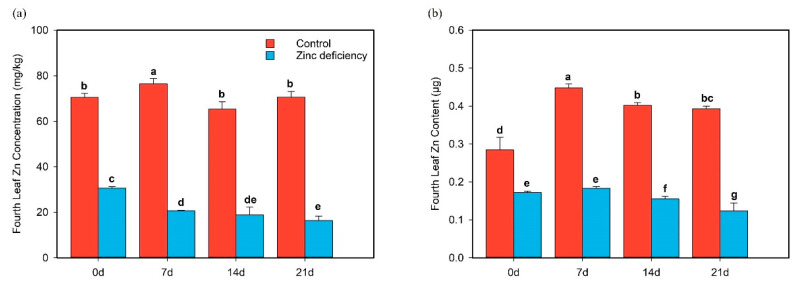
Content and concentration of Zn ions in the fourth leaf of rice seedlings under two treatments at four time points. The hydroponic plants were cultured under the condition of zinc deficiency (0 μM Zn) and control (0.75 μM Zn) when rice was in the two-leaf phase. The first sampling point was denoted as “0 d” when the fourth leaf was fully developed and then sampled every 7 days for four time points. (**a**) The concentration of zinc ions in the fourth leaf and (**b**) the content of Zn ions in the fourth leaf. Red all represents control, and blue all represents zinc deficiency. The bar chart shows the mean (+/− standard deviation) of the 15 plants in each treatment. Letters indicate statistical differences according to Student’s *t*-test (*p* < 0.05).

The content and concentration of zinc ions in the fourth leaf of Zn-deficient rice seedlings were substantially lower than those of the control. From day 0 to day 7, the concentration of zinc ions in the fourth leaf of zinc-deficient rice seedlings significantly decreased (Figure 5a), while the content of Zn ions increased slightly but not significantly (Figure 5b). The concentration and content of zinc ions increased significantly in the control treatment (Figure 5a,b).

From day 7 to day 21, the concentration of zinc ions in the fourth leaf of Zn-deficient rice seedlings gradually decreased, and the content of Zn ions on day 21 decreased significantly by 42.2% compared with day 7 (Figure 5a,b). The concentration of zinc ions in control plants also showed a downward trend, but the content of Zn ions decreased by only 14% compared with day 7.

### 2.5. Transcriptome Response to Zn Deficiency

**Figure 6:**
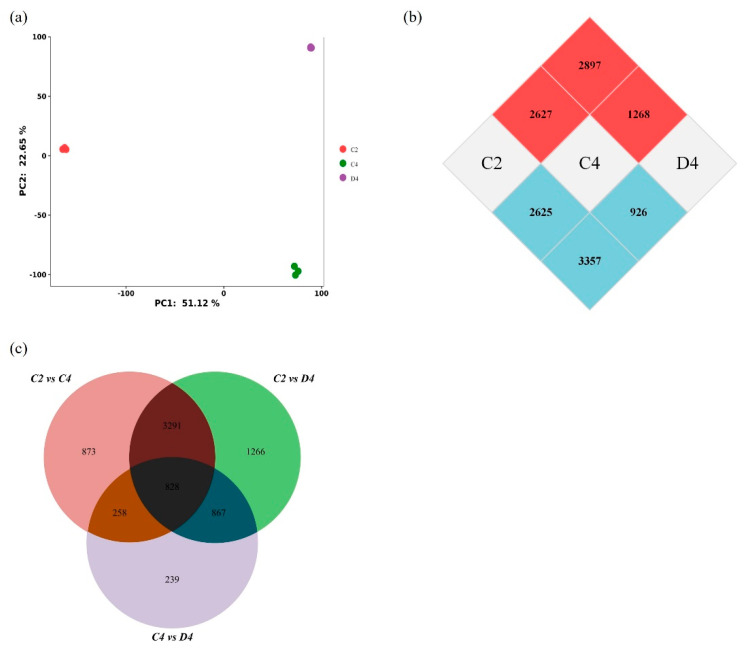
RNA sequencing analysis of the fourth leaf of rice under control and zinc (Zn) deficiency conditions. Data for each treatment were obtained from three biological replicates (eight plants per replicate). (**a**) Principal component analysis (PCA) of fourth leaf expression data. (**b**) Number of DEGs at the three selected time points. The selected time points are labeled in the central white cell, and DEGs identified in the comparison of the two time points are labeled in cross-cells with the number of upregulated (red) and downregulated (blue) regulatory genes. For example, 2897 and 3357 genes were upregulated and downregulated, respectively, in the Zn-deficient group at the fourth time point (D4) compared with the control group at the second time point (C2). (**c**) DEGs in the three comparison groups are represented with circles; the circles overlap according to the common and unique DEGs that appear, and the number above the circle and different colors represent the number of DEGs.

Principal component analysis of the RNA-Seq data showed that the variance in gene expression between biological replicates was very low. The three sample groups were grouped separately (Figure 6a). PCA separation between the samples was clear along PC1. The fourth leaf sample at the second time point of the control group is located on the left side of the x-axis, whereas the fourth time point of the control and Zn-deficient groups is located on the right side of the x-axis (Figure 6a), indicating that aging was the main component.

DEGs were identified (adjusted *p* < 0.05, log2[fold change] > 1). Among the samples at the four time points in the two treatments, those at three time points were selected for comparison. The three comparisons included comparing the second point of the control group (C2) with the fourth point of the control group (C4), as well as both of these groups with the fourth point during Zn deficiency (D4) (C2 vs. C4, C2 vs. D4, and C4 vs. D4). A total of 7622 unique DEGs were identified in the three comparison sets. In total, 828 genes were common among the three comparisons (Figure 6c). C2 and D4 mobilized the most DEGs, whereas C4 and D4 mobilized the least (Figure 6b,c). C2 vs. C4 and C2 vs. D4 had a high DEG overlap (Figure 6c). In addition, a large number of transcription factors were found in C2 vs. C4 and C4 vs. D4, mostly WRKY, NAC, MYB, bHLH, and bZIP families.

### 2.6. Biological Pathway Involvement in Zn Deficiency Treatment

**Figure 7:**
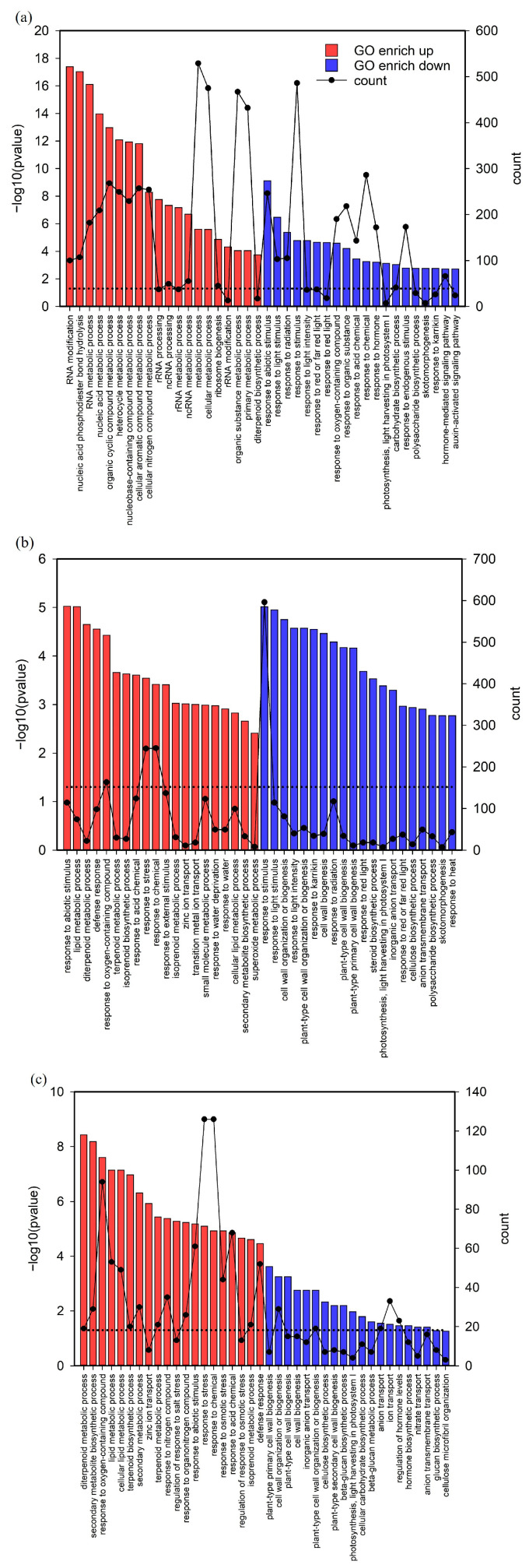
Gene enrichment analysis between three time points in the fourth leaf of rice under zinc (Zn) deficiency and control conditions. C2 refers to the second time point in the control group, C4 refers to the fourth time point in the control group, and D4 refers to the fourth time point in the Zn deficiency group. The figure presents statistically enriched (adj. *p* < 0.05) biological pathways (BPs) among both upregulated and downregulated genes in three comparisons: (**a**) C2 vs. C4, (**b**) C2 vs. D4, and (**c**) C4 vs. D4. The bar graph indicates the degree of enrichment (converting *p*-values to −log10), the red (up) and blue (down) colors indicate the direction of regulation of BP-related genes, the dots on the broken line represent the number of genes per enrichment (count), and the black dashed line indicates the −log10 values at *p* = 0.05.

The upregulated and downregulated DEGs were subjected to gene ontology (GO) enrichment analyses. Some biological processes were overexpressed in all three sets of comparisons (Figure 7). In the fourth leaf of normally aged rice (C2 vs. C4), the expression of biological pathways (BPs) related to RNA metabolism in the DEG was strongly overexpressed in the upregulated genes. Over-enrichment of BP in response to stimuli, photosynthesis, and light was observed and driven by downregulated genes (Figure 7a). C2 in the control group and D4 in the Zn-deficient group mobilized more BPs. BP enrichment driven by the upregulated genes was primarily related to oxidoreductase activity, metabolism, stress, and metal transport reactions. The BPs were driven by downregulated genes mainly involved in cell wall synthesis, light response, and photosynthesis (Figure 7b). Moreover, compared with C4, many D4-enriched BPs were related to stimulus-response, lipid metabolism, and Zn ion transport, which were all processes driven by upregulated genes. BPs driven by downregulated genes related to photosynthesis and cell wall biosynthesis were also abundant (Figure 7c). The most striking observation was the enrichment of the “zinc ion transport” BP, which was higher in C2 and D4 than in C2 and C4. This finding may be because C2 and D4 mobilized many BPs related to lipid metabolism (Figure 7).

### 2.7. Zn Ion Transport Genes Are Regulated Differently under Different Conditions

**Figure 8:**
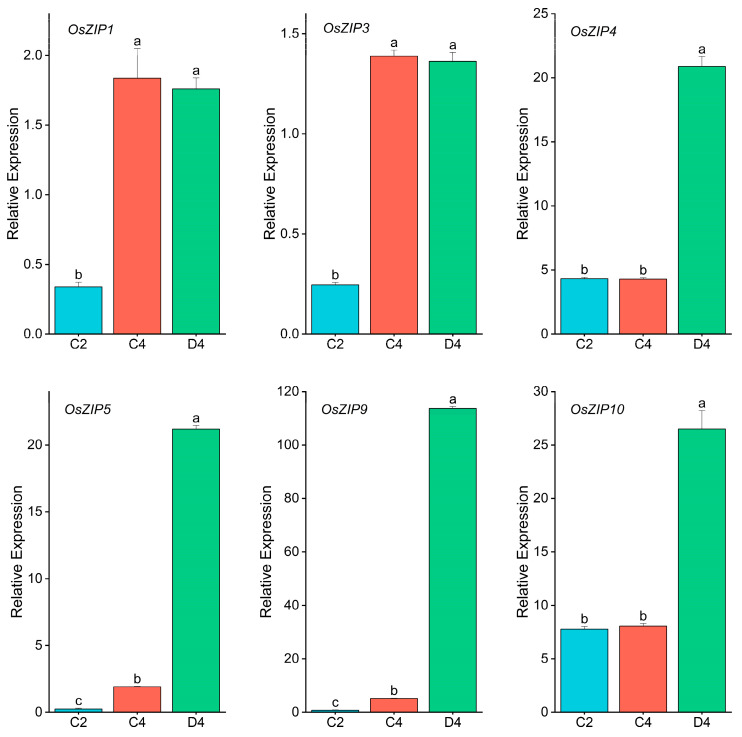
Relative expression of zinc transport gene in the fourth leaf of rice with zinc deficiency and control senescence. Quantitative real-time polymerase chain reaction (RT-PCR) transcription levels of OsZIP1, OsZIP3, OsZIP4, OsZIP5, OsZIP9, and OsZIP10 genes in the fourth leaf were compared. Red all represents C4, green all represents D4, and blue all represents C2. The bar chart shows the average (+/− standard deviation). Expression level relative to the EP. The experiment consisted of three biological replicates. The letters represent statistical differences in the one-way Analysis of Variance (ANOVA) (*p* < 0.05).

Among the 7622 DEGs (Figure 6), 10 were associated with Zn ion transport (Table 1). Among the ten genes, eight were differentially expressed in the comparison between C2 and C4, and six of these eight genes were also differentially expressed in the other two comparison groups. Two genes were differentially expressed only in the comparison between C2 and D4, as well as between C4 and D4 (Table 1). These 10 genes belonged to the metal transporter family (ZIP (six genes) and MT (four genes)). In addition, we conducted Quantitative real-time polymerase chain reaction (RT-PCR) for these six ZIP family genes to analyze the expression abundance of these six ZIP family genes in C2, C4, and D4 (Figure 8). The results showed that the expression levels of OsZIP1 and OsZIP3 in C4 and D4 were significantly higher than those of C2, but there was no significant difference between C4 and D4. The expression levels of OsZIP4 and OsZIP10 increased significantly only in D4. In D4, both OsZIP5 and OsZIP9 were significantly induced. This result was consistent with the expression in transcriptome analysis (Table 1).

## 3. Discussion

The negative inhibitory effect caused by Zn deficiency gradually strengthened in the shoot, and shoot biomass accumulation in Zn-deficient rice slowed. In addition, Zn deficiency reduced the fresh and dry weights of the fourth leaf, accelerated senescence, and shortened the growth cycle of the fourth leaf. It is worth noting that on day 0 and day 7, although the shoot fresh weight of the control treatment was higher than that of the zinc deficiency treatment, there was no significant difference. In addition, the difference in shoot fresh weight between the two treatments on day 0 was similar to the difference in shoot fresh weight between the two treatments on day 7. However, the fresh and dry weights of the fourth leaf in the Zn deficiency treatment were considerably lower than those of the control treatment. Normally, with the extension of treatment time, the difference between the two treatments should gradually increase, but this phenomenon did not appear from day 0 to day 7, and in this experiment, it was also found that the fresh weight and dry weight of the fourth leaf of zinc deficiency treatment on day 7 were significantly lower than that of the control. Therefore, there must be some factors that lead to the reduction of differences. Many studies have confirmed that nutritional deficiency can accelerate flowering [32]; Arabidopsis flowering increases stem size and leaf number [33], and considerable inhibition of vegetative growth is associated with an increase in the number of leaves in Brachypodium [34]. Plants adjust their allocation of leaf area and biomass accumulation to optimize nutrient utilization efficiency in the shoot and maintain photosynthesis [35]. A zinc deficiency experiment with Brachypodium also found that the number of leaves increased in the zinc deficiency group [34]. Therefore, we speculated that in this experiment, it may be due to the fact that under the condition of zinc deficiency, rice will accelerate the increase of leaf number by regulating the total leaf biomass and growth cycle so as to maintain photosynthesis as much as possible.

The rice root system is a whisker root system. After rice seeds germinate, the radicle extends down to form seminal roots with only one radicle [36]. In this study, seminal root senescence was inevitable, and the seminal roots only had a transitional function. With rice seedling development, seminal root growth and function weaken, and the seminal root gradually withers [37]. Seminal root length does not decrease over time. Nevertheless, this study observed a decrease in seminal root length, which can be attributed to one factor: seminal root plays a major role in the early stage of seedings development [37], but with the development of node roots, seminal roots gradually age, and node roots begin to play a major role. Consequently, the observed decrease in seminal root length and biomass may be due to the wiggle of the root system in the nutrient solution, resulting in the shedding of the aged seminal root front end. Additionally, due to the influence of zinc deficiency, the aging rate of zinc-deficient seedlings’ seminal roots is faster, and the decline rate of length and biomass is faster than that of the control. Nodal roots, or adventitious roots that grow from the base of the plant stem nodes (including tiller nodes), are the primary parts of the rice root system. In addition, node roots provide mechanical stability to plants [38]. In Zn-deficient soils, reduced or absent rhizomes adversely affect nutrient uptake in various crops, further increasing yield losses [39]. Therefore, understanding the changes in the distribution of node roots in response to Zn in rice is crucial. During Zn deficiency, the number and length of node roots were considerably reduced, and a positive correlation between nodal root number and nutrient uptake (including N, P, Fe, and Zn) has been found in rice and wheat [40]. These results indicate a strong positive correlation between root elongation, development, and nutrient uptake. In this study, the inhibitory effect on nodal roots was found to be enhanced with prolonged zinc deficiency. In the period of 7–21 days after the first sampling, there was no increase in the number of nodal roots, which is consistent with the conclusion of Sahand Amini et al. [34] that new node root development has a specific demand for zinc. In this study, the zinc-deficient rice seedlings were not exposed to exogenous zinc throughout the experiment, indicating that the early emergence and development of node roots may be associated with zinc in the seeds’ own nutrient reserve.

Zn deficiency considerably reduced the concentration and content of zinc ions in the leaves. From day 7 to 21, the fourth leaf was in senescence, and biomass decreased. Under normal aging conditions, the transfer rate of Zn ions in the fourth leaf slowed. However, when affected by Zn deficiency, the aging and Zn ion transfer rates accelerated. However, the accelerated Zn ion transfer cannot simply be regarded as a result of Zn deficiency, as leaf senescence is not a simple, continuous, and unregulated death process; moreover, leaves undergo stringent and orderly changes during aging [41,42,43]. These changes are divided into several stages (primarily the initiation, reorganization, and termination stages) [44]. The regulation of leaf senescence at different stages shows different physiological and biochemical characteristics, which is supported by the results of the gene enrichment analysis in this study. Thus, although Zn deficiency leads to accelerated Zn ion transfer, it also accelerates aging. In other words, Zn deficiency may not directly lead to the accelerated transfer rate of Zn ions. Nevertheless, the accelerated transfer may be due to the accelerated aging rate during Zn deficiency, which causes the aging process of Zn-deficiency-treated leaves to be faster than that of normal aging leaves.

Under Zn deficiency treatment, compared with the fourth leaf at the first time point, the biomass at the second time point increased considerably; however, the concentration of zinc ions decreased, likely because the roots could not obtain Zn ions from the nutrient solution. In addition, Zn deficiency reduces the total biomass and dry weight of leaves and accelerates leaf senescence and nutrient transfer rates [45]. However, the fourth leaf was still accumulating biomass at the first two time points, but the content of Zn ions did not increase. We speculate that this may be an active measure formed by rice to cope with Zn-deficiency stress. Rice optimizes the efficiency of nutrient utilization, adjusts its nutrient distribution [35], and prioritizes Zn ions and other nutrients in the latest leaves to ensure normal growth, reduce the damage caused by Zn-deficiency stress, and maintain photosynthesis. However, the latest leaves did not receive sufficient nutrients, leading to shorter growth cycles and smaller leaf areas. The sample was taken when the fourth leaf was fully deployed and still accumulating biomass; therefore, due to the growth demand of the latest leaf (the fifth leaf), the Zn ions of the last leaves would be supplied to the fifth leaf, while the supply to the fourth leaf would decrease to a certain extent. Additionally, 299 TFS from different families were identified during normal aging (C2 vs. C4), including WRKY, NAC, MYB, bHLH, and bZIP. During the Zn-deficient senescence (C2 vs. D4), 348 TFS from different families were present, including WRKY, NAC, MYB, bHLH, and bZIP.

We observed that Zn deficiency accelerates leaf aging. Leaf senescence is a necessary process that affects crop yield and quality [46]. When nutrients in the soil are exceptionally low, old leaves transform to support the growth of new organs [47], and nutrient retransferring is particularly important at this time. Usually, nutrient transfer during leaf senescence in younger plant organs helps to improve nutrient utilization efficiency [48]. However, retransferring trace elements from leaves has received much less attention than the large amounts of elements in crops, especially when the leaves are subject to nutrient deficiencies. Moreover, crops do not always contain sufficient amounts of these trace elements to meet the dietary requirements of humans. In Arabidopsis, the contents of some trace elements, such as Cu, Mo, and Zn, were found to decrease by <40% during the leaf aging process, suggesting that these nutrients are mobilized from the aging leaves [49]. Arabidopsis AtbZIP19 and AtbZIP23 are the best-studied central regulators that coordinate the Zn deficiency response in plants [50]. In rice, OsbZIP48 (basic leucine zipper 48) has the highest similarity to AtbZIP19 and AtbZIP23, key transcription factors in the Arabidopsis Zn deficiency response. OsbZIP48 is a conserved functional homolog of AtbZIP19 and AtbZIP23 [47]. OsbZIP48 is highly expressed in rice seedling leaves [51], and deleting OsbZIP48 can lead to growth arrest and, eventually, death during early development [52]. However, in the present study, OsbZIP48 was not regulated under normal or Zn-deficient aging conditions. Lilay et al. [51] investigated the role of OsbZIP48 in response to Zn deficiency in rice roots and found that this gene did not respond to Zn deficiency. Therefore, some F-bZIPs induced by Zn deficiency in wheat can achieve sustained adaptive responses in long-term Zn deficiency, with minimal transcriptional induction during Zn deficiency [53]. We found that eight genes were involved in Zn ion transfer during leaf aging in normally aging leaves, and four of them belonged to the ZIP family (OsZIP5, OsZIP9, OsZIP1, and OsZIP3). However, in contrast to normal aging, three additional genes (OsZIP4, OsZIP10, and OsZIP7) were expressed in addition to the eight genes involved in Zn ion transfer during Zn deficiency.

Sixteen ZIP transporters have been identified in rice [54], and seven ZIP genes were upregulated in this study (Table 1). OsZIP1, OsZIP3, OsZIP4, OsZIP5, OsZIP8, and other family members transport Zn and Fe [55,56]. OsZIP3, OsZIP7, and OsHMA2 are responsible for the preferential distribution of Zn in developmental tissues [12]. OsZIP1 is a Zn uptake transporter that induces gene expression under Zn deficiency [57,58]. OsZIP1 was upregulated in Zn-deficient and normal aging leaves in this study (Table 1). The results of RT-PCR analysis further verified that OsZIP1 may be involved in Zn ion retransfer during normal leaf senescence and senescence under zinc deficiency conditions (Figure 8), but ZIP1 overexpression may reduce Zn, Cu, and Cd concentrations in rice [58]. Similar to OsZIP1, OsZIP5 is upregulated during Zn deficiency and normal leaf senescence; however, OsZIP5 has a higher expression level than OsZIP1 during the aging process of zinc-deficient leaves (Figure 8). In addition, OsZIP5 is also induced by other bivalent metal ions, and the expression of OsZIP5 in stems and roots is increased under zinc deficiency induction. However, Fe and Mn deficiency only induces increased expression levels in the roots [59]. OsZIP9 was primarily expressed in the root system, with minimal expression in other parts [60]; however, in this study, OsZIP9 was upregulated in the three group comparisons (Table 1). OsZIP9 is a major influx Zn transporter in outer epidermal cells, and its overexpression considerably increases Zn and cadmium (Cd) accumulation in aboveground tissues and grains [9,27,61]. Combined with the expression level of RT-PCR (Figure 8), it was indicated that OsZIP9 may be involved in the retransfer of zinc ions during normal leaf senescence and zinc deficiency.

OsZIP7 is indispensable in transporting Zn and Cd from woody parts to developmental tissues and grains of rice [62]. Furthermore, during Zn deficiency, OsZIP7 is upregulated in buds [12]. OsZIP7 overexpression resulted in constant Zn and Cd concentrations in roots and nodes but decreased concentrations in stems and seeds [51,63]. In this study, no upregulation of OsZIP7 was found. Although OsZIP10, a homologous gene of OsZIP7, is not upregulated in normal rice leaf senescence (Table 1), and the expression level of RT-PCR also shows no difference between C2 and C4 (Figure 8), OsZIP10 is significantly upregulated in Zn-deficient leaf senescence and is involved in the retransfer of zinc ions in leaves. In addition, the loss of OsZIP10 function reduces concentrations of zinc and iron ions in rice [51].

OsZIP3 was upregulated in normally aged leaves in this study, indicating that it can transfer Zn ions during leaf aging. In addition, OsZIP3 was upregulated during leaf aging with Zn deficiency. This result was consistent with the expression level of RT-PCR (Figure 8). However, its upregulation during aging with Zn deficiency may not be simply induced by Zn deficiency. OsZIP3 was not induced by zinc deficiency and high zinc [55,56]. OsZIP3 upregulation in Zn-deficient leaves during senescence may be due to enhancing senescence via Zn-deficiency stress. In addition, OsZIP3 is not only involved in zinc transport but also involved in zinc distribution. In particular, it can unload zinc from the enlarged vascular xylem of the xylem, which is the first step for the preferential distribution of Zn to the developmental tissues. OsZIP3 is a vital transporter that regulates Zn distribution, but it cannot transport Zn alone, and heavy metal ATPase 2 is required [55].

OsZIP4 is only related to Zn transport and redistribution in rice and is primarily expressed in the vascular bundle and mesophyll cells of leaves, phloem of stems and roots, and meristem and roots [54]. In this study, OsZIP4 expression was not upregulated in typical aging leaves but was upregulated in the other two groups (Table 1), which was consistent with the expression level of OsZIP4 in RT-PCR (Figure 8), indicating that OsZIP4 transferring Zn ions in leaves may require Zn deficiency to be induced. In addition, OsZIP4 expression was increased in old and new leaves under Zn deficiency, but the expression level was higher in new leaves [54]. Therefore, OsZIP4 was more likely to be expressed in young leaves under Zn-deficient conditions.

## 4. Materials and Methods

### 4.1. Plant Material and Growth Conditions

The rice variety Zhonghua 11 (Jiangsu, Baige Gene Technology Co., Ltd.) was used in this experiment. The seeds were disinfected with 50% sodium hypochlorite solution for 45 min. After five washings with deionized water, the seeds were immersed in deionized water in the dark for 2 days at 38 °C. Subsequently, the seeds germinated in the dark at 25 °C on wet filter paper (wet with deionized water) for two days. After germination, the seedlings were transplanted into a hydroponic box containing deionized water for two weeks. Then, deionized water was replaced with the International Rice Research Institute (IRRI) formula nutrient solution, and the culture was continued under control or treatment conditions. Two static conditions were used: control (0.75 μM Zn) and Zn deficiency (0 μM Zn). After 12 days of treatment, the first sample was taken (at which time the fourth leaf was fully unfolded) and recorded as 0 days, followed by sampling every 7 days for a total of 4 times. The fourth leaf and the root systems were sampled. The samples were collected for (i) root and leaf phenotypic analyses, (ii) ion analysis, and (iii) RNA sequencing. The nutrient solution was replaced every 3 days, and the pH was adjusted to approximately 5.4. Harvesting took place 2 h after the beginning of the afternoon. The growth conditions were 14 h of light at 28 °C followed by 10 h of darkness at 20 °C. During all experiments and conditions, the hydroponic boxes and solution containers were washed prior to use with 6N hydrochloric acid to remove Zn traces.

### 4.2. Ion Analysis

Using inductively coupled plasma mass spectrometry (ICP-MS), the concentration and content of zinc ions in the fourth leaves of rice were determined. The fourth leave of rice under control and zinc-deficient treatments was sampled at four time points, with three replicates each. The samples were dried at 65 °C for 4 days. After drying, the samples were ground, and 10 mg was weighed into an EP tube that had been cleaned and dried. The EP tube was washed twice with ultrapure water, using 2 mL each time, and then transferred to a digestion vessel. In the digestion vessel, 5 mL of concentrated nitric acid and 2 mL of hydrogen peroxide were added. The mixture was digested until clear and then heated on an electric heating plate to drive off the acid. The solution was then transferred to a 10 mL volumetric flask, filtered, and analyzed for zinc element [64].

### 4.3. Quantitative RT-PCR

Upon harvest, the fourth leaves were snap-frozen in liquid nitrogen and stored at −80 °C. Total RNAs were extracted from the fourth leaves samples, and cDNA preparation and quantitative RT-PCR were according to the description. Relative transcript level normalization was performed with the 2^−ΔΔCt^ method using EP (LOC_Os05g08980) reference genes. Primer pairs are shown in Table 2.

### 4.4. RNA Sequencing

After sampling, the tissues were frozen in liquid nitrogen and stored at −80 °C. Nine samples were used (tissue: a fourth leaf; the second time point of the control group was used as the control and compared with the fourth time point of the control and Zn-deficient groups with three replicates). Total RNA was extracted, and the RNA quality was tested. Only RNA of good quality was constructed for subsequent libraries. The detection of RNA samples primarily included the following methods: (1) agarose gel electrophoresis to analyze RNA degradation and whether there was contamination, and (2) RNA purity was measured using a Nanodrop (OD260/280 ratio). After the samples were qualified, oligo (dT) beads separated the mRNA, which were then divided into short fragments by adding an interrupting reagent. The resulting fragments were used as a template to synthesize the first cDNA strand, and the Second Strand Synthesis Mix was added to synthesize the second strand. The RNA-Seq adapter was connected, and the connected products were purified for PCR amplification. The 300–600 bp target products were purified using magnetic beads for sequencing. After library construction was completed, a Qubit was used for preliminary quantification. The library was then diluted to 1 ng/uL, and a Qseq100 DNA Analyzer was used to detect the insert size of the library. Quantitative PCR was used to accurately quantify the effective concentration of the library (effective concentration > 2 nM) to ensure its quality. After library inspection, different libraries were pooled according to the requirements of effective concentration and the target amount of data, and the libraries were sequenced on the Illumina NovaSeq 6000 platform using the PE150 sequencing strategy. Raw data were filtered using fastp software v.0.23.4, which includes the following steps: (1) primers and adaptor sequences were removed; (2) removal of sequences with fragment lengths <50 bp; (3) removal of the N base that reached a certain proportion of reads (default: 5 bp); (4) low-quality bases with mass values <20 were removed; and (5) four bases were used as windows to calculate the average base mass. The base was removed if the average base mass was <20 (Q20). Hisat software v.20.6 was used to map the similarity between clean data and reference genomes. The overall mapping rate of the nine groups was >96%.

### 4.5. Data Analysis

edgeR v3.42.4 was used for differential expression gene (DEG) analysis. The DEGs in the standard were as follows: Calculated expression ratio change (fold change) met |log2(fold change)| > 1; 2. The Q value was obtained by FDR correction of the *p* value, and both *p* value ≤ 0.05 and Q (FDR) value ≤ 0.05 were satisfied. Genes above the criteria were considered differentially expressed and used for principal component analysis (PCA). The clusterProfiler in R v.4.8.3 was used for GO enrichment analysis of differential genes with an adjusted *p* value of ≤0.05. Other statistical tests were conducted using analysis of variance or Student’s *t*-test.

## 5. Conclusions

In summary, zinc deficiency inhibited the growth and development of rice shoots and roots to varying degrees. In the zinc deficiency treatment group, the senescence and zinc ion transport rates of leaves were found to be greatly accelerated. In addition, through RNA sequencing analysis, zinc deficiency regulated more BPs, and zinc ion transport BP was particularly evident. In zinc-deficient treatment, more ZIP family members regulated the function of transporting zinc ions during leaf aging, and OsZIP4 and OsZIP10 were only upregulated in the Zn-deficient treatment group. Future studies are needed to validate and explore the potential information of zinc ion transport genes in leaves.

## Figures and Tables

**Table 1:** Upregulated zinc-ion-transport-related genes in differentially expressed genes (DEGs).

Rice			
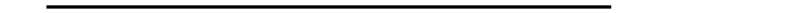			
Gene ID	Gene Description	Gene Name	Inclusion Contrast
Os12g38300	Metallothionein-like protein 4C	OsMT1c	C2_vs._C4; C2_vs._D4; C4_vs._D4
Os05g39560	Zinc transporter 5-like	OsZIP5	C2_vs._C4; C2_vs._D4; C4_vs._D4
Os12g0571000	Metallothionein-like protein 4B	—	C2_vs._C4; C2_vs._D4; C4_vs._D4
Os05g39540	Zinc transporter 9-like	OsZIP9	C2_vs._C4; C2_vs._D4; C4_vs._D4
Os12g38051	Metallothionein-like protein 4B	OsMT-I-4b	C2_vs._C4; C2_vs._D4; C4_vs._D4
Os12g0567800	Metallothionein-like protein 4C	—	C2_vs._C4; C2_vs._D4; C4_vs._D4
Os01g74110	Zinc transporter 1-like	OsZIP1	C2_vs._C4; C2_vs._D4
Os04g52310	Zinc transporter 3-like	OsZIP3	C2_vs._C4; C2_vs._D4
Os08g10630	Zinc transporter 4-like	OsZIP4	C2_vs._D4; C4_vs._D4
Os06g37010	Zinc transporter 10	OsZIP10	C2_vs._D4; C4_vs._D4

**Table 2:** Primers designed and used for quantitative RT-PCR.

Gene	Forward Primer (5′–3′)	Reverse Primer (5′–3′)	Primer Efficiency
OsZIP1	TGAGCAAAATGGTGGAAAGC	CAGTTGCAACCCCTGTATGA	1.972
OsZIP3	TTGCGGGCCTTGTTTCAATG	GCACCAACTAGTGGCCTGAT	1.895
OsZIP4	TCGGTGATCATCGGGGTTTC	AATCTGCAGCGAGGAGATCG	1.985
OsZIP5	TGGTGCACTCGCTCATCATC	GTCCCCTTCTCTGCACCTTG	1.977
OsZIP9	CACTAGGTGCATCCGAGAGC	GCTGATGATGAGCTGCAACC	1.946
OsZIP10	CCTATCGTTGGGCGTCTCTC	CACTTGAAGCCTTGGGTTGC	1.821
EP	TGAGCAAAATGGTGGAAAGC	CAGTTGCAACCCCTGTATGA	1.981

## Data Availability

The original contributions presented in the study are included in the article; further inquiries can be directed to the corresponding author.
